# Anca negative pauci-immune crescentic glomerulonephritis and mixed connective tissue disease: a case study

**DOI:** 10.1590/2175-8239-JBN-2019-0003

**Published:** 2019-03-18

**Authors:** Sara Fernandes, Catarina Teixeira, Luis Pedro Falcão, Ana Cortesão Costa, Mário Raimundo, Sónia Silva, João Cardoso, Edgar De Almeida

**Affiliations:** 1 Hospital Beatriz Angelo, Loures, Portugal.; 2 Centro Hospitalar Universitário Lisboa Norte, Lisboa, Portugal.

**Keywords:** Glomerulonephritis, Mixed Connective Tissue Disease., Glomerulonefrite, Doença Mista do Tecido Conjuntivo.

## Abstract

One of the most common causes of rapidly progressive glomerulonephritis (RPGN) is pauci-immune crescentic glomerulonephritis (CrGN). In the majority of cases, this condition has a positive serologic marker, the anti-neutrophil cytoplasmic antibodies (ANCAs), but in approximately 10% there are no circulating ANCAs, and this subgroup has been known as the ANCA-negative pauci-immune CrGN. RPGN can be associated with systemic diseases, but there are only few case reports describing the association with mixed connective tissue disease (MCTD). The authors report a case of ANCA-negative CrGN associated with a MCTD.

## Resumo

Uma das causas mais comuns da glomerulonefrite rapidamente progressiva (GNRP) é a glomerulonefrite crescêntica (GNC) pauci-imune. Na maioria dos casos, a patologia apresenta um marcador sorológico positivo, o anticorpo anticitoplasma de neutrófilos (ANCA), mas em cerca de 10% dos pacientes não há ANCAs circulantes, perfazendo um subgrupo da patologia conhecido como GNC pauci-imune ANCA-negativa. A GNRP pode estar associada a doenças sistêmicas, mas são poucos os relatos de caso que descrevem sua associação com doença mista do tecido conjuntivo (DMTC). O presente artigo relata um caso de GNC ANCA-negativa associada a DMTC.

## INTRODUCTION

Pauci-immune crescentic glomerulonephritis (CrGN) is the most common cause of rapidly progressive glomerulonephritis (RPGN) in adults.^1^^,^ 2

Mostly, CrGN is attributed to systemic small-vessel vasculitis, like granulomatosis with polyangiitis, microscopic polyangiitis and eosinophilic granulomatosis with polyangiitis, but also with renal limited vasculitis in a small number of cases. CrGN and RPGN share a common serologic hallmark, defined by the presence of the anti-neutrophil cytoplasmic antibodies (ANCAs), and for this reason they are known as ANCA-associated vasculitis. However, there is a small group of pauci-immune CrGN without ANCA positivity, representing about 10 % of cases.^1^^,^ 3 Only few cases of ANCA-negative pauci-immune CrGN have been published, so data available on this subset of patients is limited.^1^ Most of these cases are idiopathic and not associated with connective tissue diseases.^3^ The mixed connective tissue disease (MCTD) is defined as a syndrome that shares features of systemic sclerosis, polymyositis, and systemic lupus erythematosus (SLE). A laboratory characteristic of this syndrome is a high titter of anti- ribonucleoprotein (RNP) antibodies and positive antinuclear antibodies (ANA) with high titter speckled pattern. The antibodies to double-stranded deoxyribonucleic acid (dsDNA) Sm, Ro and La might also be present, although not dominant or persistent. Renal involvement in MCTD is less common than in typical SLE. Normally it occurs either as a membranous nephropathy, less frequently as mesangioproliferative glomerulonephritis or renal vasculopathy of scleroderma.^4^ Pauci-imune CrGN is a rare form of renal involvement in MCTD and is sparsely reported. The authors aim to describe a case of a pauci-immune CrGN, with negative ANCA, which was associated with MCTD.

## CASE REPORT

A 58-year-old black man with history of hypertension, interstitial lung disease of unclear etiology, and recurrent right pleural effusion was admitted at our institution. The respiratory disease was attributed to an occupational exposition to tar and concrete, but definitive diagnosis could not be established. His outpatient medications were esomeprazole, hydrochlorothiazide plus valsartan, fluticasone, and salmeterol. 

He was admitted at the emergency department with anorexia, asthenia, and right pleuritic thoracalgia lasting for two weeks. There was no history of new medications. He mentioned an episode of polyarthralgia and edema of lower extremities one year before, which had a spontaneous resolution. On admission, he was afebrile and his blood pressure was 170/91mmHg. He had diminished pulmonary sounds on the inferior lobes. The presence of puffy hands was notorious. The rest of his physical examination was unremarkable.

Initial investigation revealed a normocytic and normochromic anemia (hemoglobin 11 g/dL), C reactive protein of 0.84 mg/dL, erythrocyte sedimentation rate of 89 mm/h, blood urea 114 mg/dL, and serum creatinine of 3.5 mg/dL. Urinalysis presented with hematuria and proteinuria and an urinary sediment with numerous red blood cells (10-30 per high power field) and rare leucocytes (<5 per high power field). Chest radiography showed bilateral pleural effusion. Mild increased parenchymal echogenicity was found in the renal ultrasound with no dilation of the urinary system. Normocytic and normochromic anemia (Hb 11g/dL), normal serum creatinine of 0.98mg/dL and a normal urinalysis were present in laboratory analysis two years before. A RPGN was suspected and extended investigation showed a 24 hours urinary protein excretion of 5.9g, hypoalbuminemia of 2.8g/dL, hypercholesterolemia (total cholesterol 228 mg/dL and LDL 153 mg/dL), and hypertriglyceridemia (298 mg/dL). A strongly positive ANA (1/1280) with speckled pattern and positive anti-RNP antibodies was apparent but the remaining immunologic studies were negative, including ANCA, anti-glomerular basement membrane antibodies, anti-dsDNA, anti-La, anti-Ro, and anti-Sm. The complement levels (C3 and C4) were within the normal range and serum electrophoresis excluded a monoclonal gammopathy. Peripheral blood cultures were sterile. Tests for human immunodeficiency virus, hepatitis C virus, and hepatitis B virus were negative.

**Figure 1:**
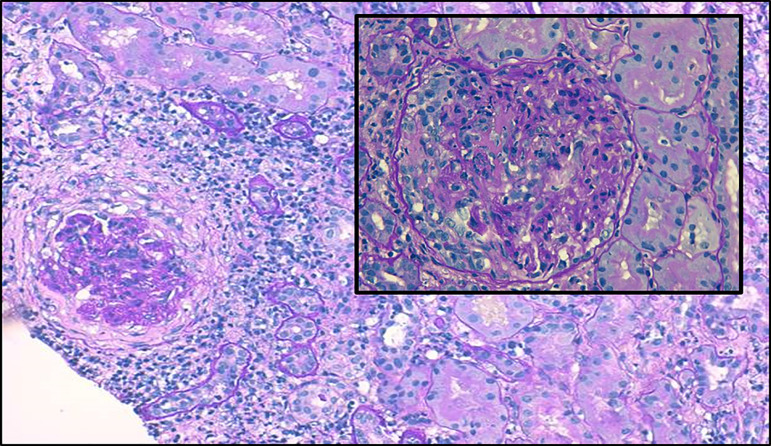
Periodic acid-Schiff staining shows a cellular crescent, with cellular inflammatory reaction, mainly mononuclear. Most of the tubules have a preserved structure (100x). Inset showing an amplification of a glomerulus with a cellular crescent (200x).

**Figure 2:**
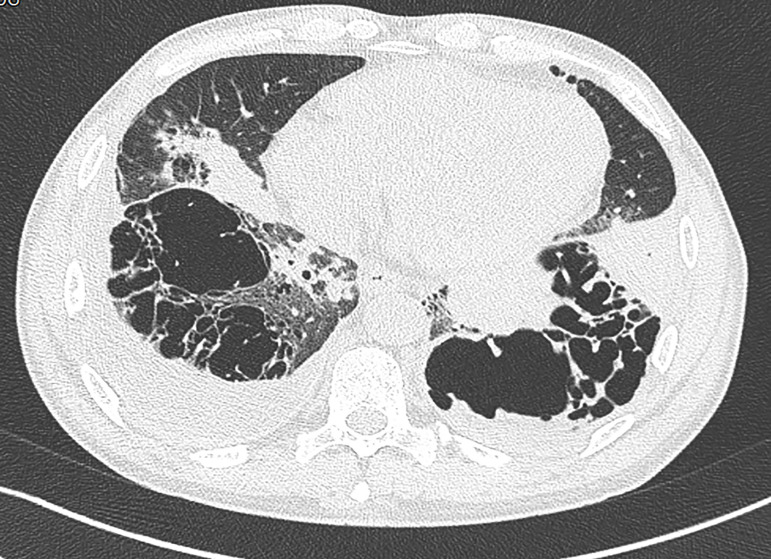
Chest computed tomography (CT): lung cavities with destruction of the lung parenchyma in the inferior lobes on both sides and a right pleural effusion.

Renal biopsy (Figure 1) revealed 10 out of 11 glomeruli with cellular crescents, some with fibrinoid necrosis and rupture of Bowman capsule. There was peri-glomerular fibro-edema, involving also 30% of the cortex. Severe inflammatory infiltrate, mainly mononuclear, was present in the interstitium. There were spots of acute tubular necrosis, but most of the tubules were preserved. Immunofluorescence was negative. Electron microscopy was not performed. These changes were compatible with pauci-immune CrGN. Steroid therapy was initiated with favorable response, but given the severity of the biopsy findings, cyclophosphamide was added. As a complication of immunosuppressive therapy, a bacterial pneumonia was successfully treated. For better characterization of the severity of the respiratory disease, a chest computed tomography (CT) showed lung cavities with destruction of the lung parenchyma in both inferior lobes and a right pleural effusion (Figure 2). A pulmonary CT performed two years before already showed these cavities, which were stable in size. A bronchofibroscopy showed ulcerative lesions in superior lobar bronchus that may be suggestive of vasculitis, diffuse inflammatory signs as well as abundant secretions. Broncho-alveolar lavage was negative for *Mycobacterium tuberculosis* and other agents. At this point, a systemic disease with renal and respiratory involvement, previously present but undiagnosed, was considered, which led to a capillaroscopy revealing a secondary Raynaud phenomenon and a late scleroderma pattern. Echocardiography excluded pulmonary hypertension. Spirometry results disclosed a normal forced vital capacity (4.43 L, 99% of predicted), normal static volumes (total lung capacity of 6.61 L, 93% of predicted), normal airway resistance and a reduced diffusing capacity (DLCO 4.03 mmol/min/kPa, 40.7 % of predicted). These results were compatible with the diagnosis of MCTD. After induction therapy with cyclophosphamide, patient started on azathioprine (2 mg/Kg/day). He showed a significant improvement with serum creatinine decreasing to 1.2 mg/dL. Three months after the initial episode, the patient was asymptomatic. A continuous renal function improvement was evident (serum creatinine of 0.97 mg/dL, proteinuria of 335 mg/24 hours, with a normal urinary sediment) as well as an improvement in the respiratory symptoms and in the pulmonary function tests.

## DISCUSSION

MCTD is a rare syndrome with overlap features of rheumatic disorders, such as SLE, systemic sclerosis and polymyositis with the serologic marker of high titters of anti-RNP antibodies. The Alarcon-Segovia and Kahn's diagnostic criteria are the most used algorithms for establishing the diagnosis of MCTD^5^. Both classifications include serological (high titters of anti-RNP antibodies) and clinical (swollen hands, synovitis, myositis, and Raynaud phenomenon) criteria.^6^ This patient presented with a higher titter of anti-RNP antibodies, swollen hands, synovitis, and Raynaud phenomenon, filling the diagnosis criteria for MCTD. Although almost any organ can be involved in MCTD, severe renal involvement is infrequent and it is hypothesized that high titters of anti-RNP antibodies may protect against the development of diffuse proliferative glomerulonephritis.^7^^-^ 11 The most common presentations of renal disease in MCTD are membranous nephropathy and mesangioproliferative glomerulonephritis. Interstitial nephropathy and renal vasculopathy are less frequent and could lead to malignant hypertension as observed in scleroderma renal crisis.^9^^-^ 11 Published data reports only few cases of CrGN associated with connective tissue diseases, especially with MCTD. Considering only the subset of patients with ANCA-negative pauci-immune CrGN, the number of reported cases is even smaller.^4^^,^ 12 ^,^ 13 We could only find 3 cases of ANCA negative pauci-immune CrGN associated with a MCTD.^14^^-^ 16 Because of the rarity of this association, we decided to report a case of a patient with biopsy proven pauci-immune necrotizing CrGN in the absence of ANCA positivity that simultaneously presented clinical and serological markers of MCTD. Specific therapeutic protocols for patients with CrGN and MCTD are not available due to the rarity of this association. The treatment for MCTD should be individualized depending on organ involvement and severity.^6^^,^ 17 ^,^ 18 In this case report, the therapeutic approach was based on the most commonly accepted strategy for pauci-imune CrGN because of the magnitude of the renal involvement and included cyclophosphamide in combination with high dose steroids, followed by azathioprine.^19^ Successful use of azathioprine as maintenance therapy was reported in one case of pauci-immune CrGN associated with MCTD.^15^ Azathioprine has also been used on MCTD with good results, especially when there is pulmonary, articular, or neurologic involvement.^6^^,^ 17 ^,^ 20 As expected, the renal outcomes would have been better if the treatment started in early stages of the disease.^5^^,^ 14 A favorable clinical outcome was observed, with renal function recovery, normalization of urinary sediment, significant proteinuria reduction and substantial improvement in pulmonary function tests. This multi-organ improvement after immunosuppression consolidated the hypothesis of a common immune origin in both renal and pulmonary dysfunctions.

## CONCLUSION

This case study reports an extremely rare form of renal involvement in MCTD: an ANCA-negative pauci-immune CrGN. This report also highlighted the crucial role of detailed clinical examination, serologic markers, and an elevated level of suspicion to reveal a less frequent, and sometimes missed diagnosis. There is no treatment protocol for this condition, but careful assessment of organ involvement and severity should guide the best therapeutic strategy. In this case report, successful control of the disease was obtained with cyclophosphamide plus high dose corticosteroids followed by azathioprine.
